# γ-Tocotrienol Protects against Mitochondrial Dysfunction, Energy Deficits, Morphological Damage, and Decreases in Renal Functions after Renal Ischemia

**DOI:** 10.3390/ijms222312674

**Published:** 2021-11-24

**Authors:** Grazyna Nowak, Judit Megyesi

**Affiliations:** 1 Department of Pharmaceutical Sciences, College of Pharmacy, University of Arkansas for Medical Sciences, Little Rock, AR 72205, USA; 2 Division of Nephrology, Department of Internal Medicine, College of Medicine, University of Arkansas for Medical Sciences, Little Rock, AR 72205, USA; MegyesiJuditK@uams.edu

**Keywords:** γ-tocotrienol, renal ischemia, acute kidney injury, mitochondrial dysfunction, respiration, electron transport chain, oxidative phosphorylation, ATP synthase/F_0_F_1_-ATPase, ATP, renal cortical tubules, tubular necrosis

## Abstract

Ischemia-induced mitochondrial dysfunction and ATP depletion in the kidney result in disruption of primary functions and acute injury of the kidney. This study tested whether γ-tocotrienol (GTT), a member of the vitamin E family, protects mitochondrial function, reduces ATP deficits, and improves renal functions and survival after ischemia/reperfusion injury. Vehicle or GTT (200 mg/kg) were administered to mice 12 h before bilateral kidney ischemia, and endpoints were assessed at different timepoints of reperfusion. GTT treatment reduced decreases in state 3 respiration and accelerated recovery of this function after ischemia. GTT prevented decreases in activities of complexes I and III of the respiratory chain, and blocked ischemia-induced decreases in F_0_F_1_-ATPase activity and ATP content in renal cortical tissue. GTT improved renal morphology at 72 h after ischemia, reduced numbers of necrotic proximal tubular and inflammatory cells, and enhanced tubular regeneration. GTT treatment ameliorated increases in plasma creatinine levels and accelerated recovery of creatinine levels after ischemia. Lastly, 89% of mice receiving GTT and 70% of those receiving vehicle survived ischemia. Conclusions: Our data show novel observations that GTT administration improves mitochondrial respiration, prevents ATP deficits, promotes tubular regeneration, ameliorates decreases in renal functions, and increases survival after acute kidney injury in mice.

## 1. Introduction

Tocotrienols are members of the vitamin E family comprising four tocopherols and four tocotrienols. They show diverse biological effects, which are distinct from the effects of tocopherols [1,2,3]. Tocotrienols have an isoprenoid side chain. In contrast, tocopherols have a phytyl side chain [4]. A large number of genes are under the control of tocotrienols but not tocopherols [2,5,6]. Consequently, tocotrienols have functionally unique biological properties distinct from properties of tocopherols including activation of antiproliferative and cell death-inducing pathways in cancer cells, but protective actions in nonmalignant cells [2,7,8]. Tocotrienols are more potent antioxidants and anti-inflammatory agents compared to tocopherols and have potent neuroprotective properties that are not observed with tocopherols [6,9]. γ-Tocotrienol (GTT) is known for its anticancer, antiproliferative, and proapoptotic effects in a wide range of human cancers [10,11]. It has been postulated that antiproliferative effects of GTT are mediated through the inhibition of phosphoinositide-3 kinase (PI-3 K)/Akt- and/or nuclear factor κ-light-chain-enhancer of activated B cells (NFκB) pathways that are crucial for cancer cell proliferation [12,13]. The apoptosis-inducing actions of GTT in cancer cells involve the mitochondrial pathway and inhibition of the complexes I and II of the respiratory chain, reduction in oxidative phosphorylation, activation of Bax and down-regulation of Bcl-2, and the release of cytochrome from mitochondria [14,15,16].

In contrast to the proapoptotic effects in cancer cells, γ- and δ-tocotrienols are very potent antioxidants, anti-inflammatory, and protective agents in a variety of nonmalignant cell types [1,17]. GTT protects against ischemia and reperfusion injury in cardiomyocytes [18,19,20], H_2_O_2_-induced oxidative stress and apoptosis in astrocytes [21,22], irradiation injury to hematopoetic and gastrointestinal cells [23,24], and cigarette smoke-induced injury in lung cells [25]. Further, GTT delays premature senescence of human fibroblasts by blocking caspase-9 and caspase-3 activations, cytochrome c release from mitochondria, downregulating Bax while upregulating Bcl-2A1 mRNA expressions, decreasing Bax/Bcl-2 protein ratio, and inhibiting apoptosis [26].

GTT has potent radioprotective effects, ameliorates intestinal injury induced by irradiation, promotes recovery of the intestine after injury, decreases oxidative stress in the vasculature, and improves survival in animals after total body irradiation [23,24]. GTT-conferred protection and reduction of mortality after lethal dose of radiation is associated with accelerated recovery of hematopoietic progenitor and white blood cells including neutrophils and monocytes, platelets, and reticulocytes [23,24]. It has been suggested that GTT-conferred protection of intestinal cells against radiation-induced injury is mediated through HMG-CoA reductase, upregulation of antiapoptotic genes, and downregulation of proapoptotic genes [27,28,29,30,31,32].

It has been proposed that tocotrienols also have nephroprotective properties [33,34]. Chronic treatment of rats for 21 days with a tocotrienol-rich fraction extracted from palm oil decreased chemically (K_2_Cr_2_O_7_)-induced kidney injury by decreasing morphological damage to renal proximal tubules (RPT) and improving RPT function, glomerular filtration rate, and cellular redox status [33]. However, it is unknown which of the components of the tocotrienol-rich fraction are responsible for these effects. Ischemic injury to the kidney caused by inadequate O_2_ and nutrient delivery is a common clinical condition resulting from the failure of mitochondrial bioenergetics that leads to energy (ATP) deficits in the renal cortical tissue. RPT cells make up over 80% of renal cortex and are a major target of ischemia due to their almost total dependence on mitochondria for ATP synthesis and a very low capacity for glycolysis [35,36]. Renal ischemia greatly affects mitochondrial respiration, generation of proton motive force, and oxidative phosphorylation in RPT cells. Our previous study demonstrated that RPT cells take up and accumulate GTT [34]. We have also shown that GTT, but not α-tocopherol, administration in primary cultures of RPT cells in vitro diminishes mitochondrial dysfunction, ATP deficits, and cell death induced by an oxidant [34]. GTT improves mitochondrial respiration and coupling, maintains mitochondrial membrane potential, and decreases cell death in oxidant-injured RPT cells in vitro [34]. However, it is unknown if GTT offers similar protection to the renal mitochondria in vivo and whether GTT decreases kidney injury induced by ischemia. Thus, the objectives of this study were to test whether GTT administration: (1) ameliorates mitochondrial dysfunction and ATP deficits in renal cortical tubules; (2) targets mitochondrial proteins involved in oxidative phosphorylation; (3) reduces morphological damage to the kidney and the decline in renal functions; (4) promotes recovery of kidney function after ischemia-induced kidney injury, and (5) promotes survival.

## 2. Results

### 2.1. Treatment of Mice with GTT Improves Renal Function after Ischemia

**Figure 1:**
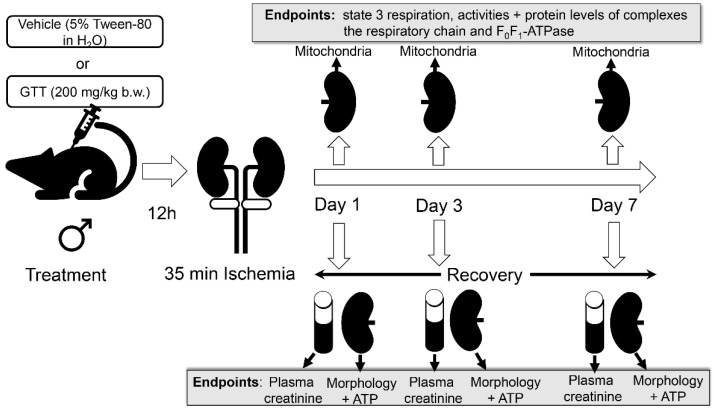
Diagram representing the experimental design and the endpoints measured in this study.

**Figure 2:**
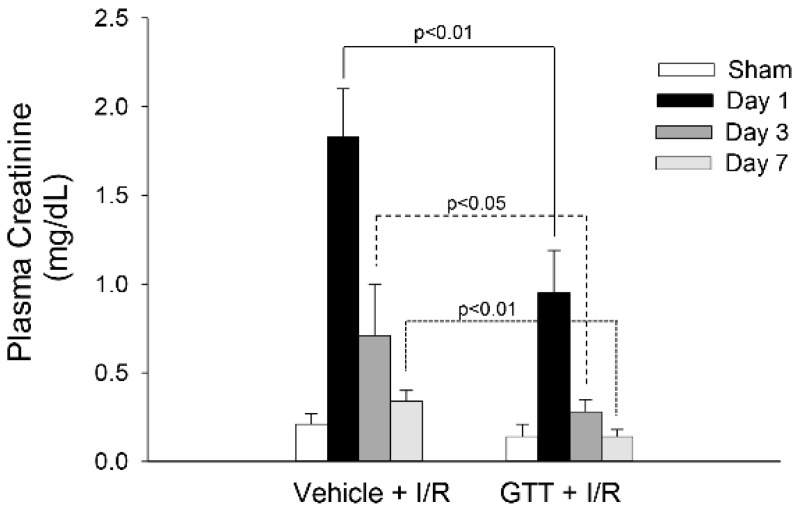
Administration of γ-tocotrienol (GTT; 200 mg/kg b.w.) reduces ischemia-induced decreases in renal function and promotes recovery of renal function after ischemia. Renal function was assessed using plasma creatinine levels collected from sham animals and at different time points during recovery after bilateral renal ischemia (35 min). Results are the average ± S.E. of data obtained from 6–13 male mice. Results statistically different (*p* < 0.05 or *p* < 0.01) from respective sham controls are marked.

The experimental design for this study, the treatments of animals, and the endpoints measured are shown in Figure 1. Treatment of mice with the vehicle (5% Tween-80 in H_2_O) had no effect on serum creatinine levels (0.18 ± 0.04 vs. 0.21 ± 0.06 mg/dL in vehicle treated vs. nontreated control mice). Ischemia induced nine-fold increases in serum creatinine levels at 24 h of reperfusion in mice treated with the vehicle (Figure 2). Serum creatinine levels in the vehicle-treated mice gradually declined after reperfusion and recovered within 7 days after ischemia (Figure 2). GTT treatment significantly reduced increases in serum creatinine levels at 24 h after ischemia in comparison with the levels observed in mice treated with the vehicle (Figure 2). Further, the levels of serum creatinine in GTT-treated mice recovered by day 3 after ischemia (Figure 2). These data show that the treatment with GTT reduces ischemia-induced decreases in renal function and promotes recovery of kidney functions after ischemia-induced acute kidney injury.

### 2.2. GTT Administration Improves Renal Morphology after Ischemia

**Figure 3:**
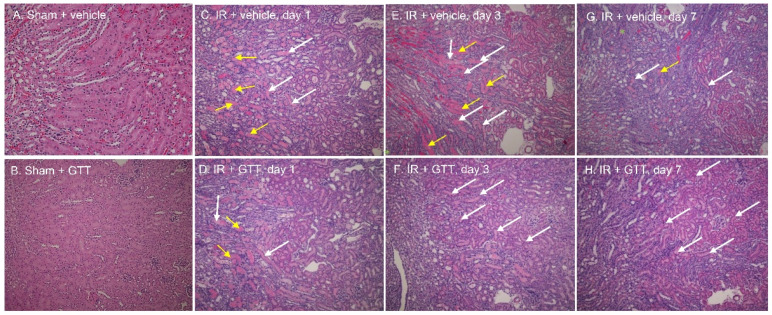
The effect of γ-tocotrienol (GTT) administration (200 mg/kg b.w.) on the morphology of the renal cortex at different time points after bilateral renal ischemia (35 min) in mice. (**A**) Sham-operated kidney ± vehicle—day 1. (**B**) Sham-operated kidney ± GTT—day 1, (**C**) Ischemia ± vehicle—day 1, (**D**) Ischemia ± GTT—day 1, (**E**) Ischemia ± vehicle—day 3, (**F**) Ischemia ± GTT—day 3, (**G**) Ischemia ± vehicle—day 7, (**H**) Ischemia ± GTT—day 7. The vehicle and GTT were administered subcutaneously 12 h prior to surgery to induce ischemia. The images are representative of three to four kidneys obtained from individual male mice used in each treatment group. The images were acquired using Nikon Eclipse E800 light microscope and Nikon Plan Apo objective (10×). Magnification × 122. Yellow arrows show necrotic proximal tubules. White arrows show regenerating tubules.

Kidneys from vehicle- and GTT-treated mice did not show any signs of morphological damage and were morphologically indistinguishable from sham-operated, untreated noninjured kidneys (Figure 3A,B). Kidneys from either vehicle- or GTT-treated mice harvested 24 h after ischemia/reperfusion surgery showed characteristic morphological damage of ischemic injury, such as severe confluent necrosis of the proximal tubules (yellow arrows), brush-border loss, and tubular cast formation (white arrows) at the cortico-medullary junction (S3 segment) (Figure 3C). This was accompanied by tubular dilatation, interstitial edema, degeneration, infiltration of inflammatory cells, and erythrocyte extravasation (Table 1). Assessment of seven criteria of kidney morphological damage revealed significant degree of injury in ischemic kidneys from vehicle-treated mice when compared to kidneys from sham-operated animals (Table 1).

The extent of ischemia-induced morphological damage, the loss of brush border, and necrosis in proximal tubules, were reduced in kidneys from GTT-treated mice (Figure 3D; Table 1). GTT treatment reduced confluent necrosis, cast formation, and the number of inflammatory cells in kidneys at 24 h after ischemia (Figure 3C,D; Table 1). The differences between morphology of vehicle- and GTT-treated kidneys were more apparent at 72 h after ischemia/reperfusion injury. There was still widespread damage in kidneys of vehicle-treated animals with very little evidence of regeneration (Figure 3E; Table 1). In contrast, kidneys from GTT-treated mice showed only mild tubular dilatation and cast formation (Figure 3F; Table 1). There was advanced morphological regeneration and the previously damaged proximal tubules were relined with new flat epithelium (Figure 3F). By day 7 after ischemia, the kidneys from mice treated with GTT were almost completely regenerated, whereas the remnants of necrosis, cast formation, and some tubular dilatation were still present in the kidneys from vehicle-treated mice (Figure 3G,H). 

These data demonstrate that treatment of mice with GTT prior to ischemia reduces kidney injury caused by ischemia and accelerates recovery of kidney morphology after injury.

### 2.3. Treatment with GTT before Ischemia Improves Survival during Reperfusion

**Figure 4:**
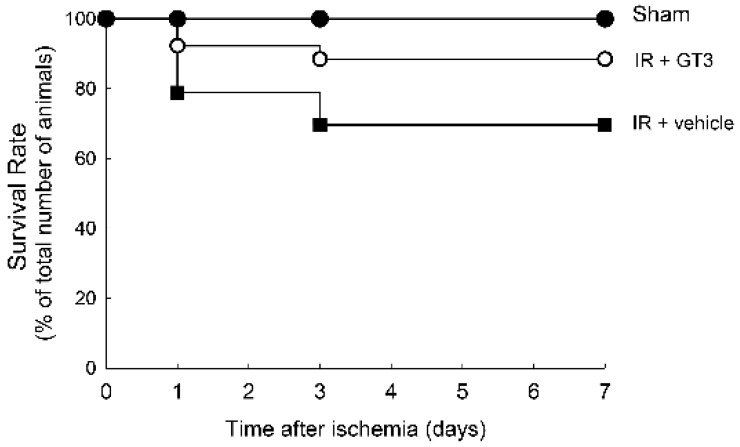
Administration of γ-tocotrienol (GTT) improves survival rate after ischemia-induced acute kidney injury. The comparison of survival of sham mice treated with the vehicle (5% Tween80) or GTT (200 mg/kg b.w. in 5% Tween 80), mice treated with vehicle followed by bilateral ischemia (35 min) and reperfusion, and mice treated with GTT followed by bilateral ischemia (35 min) and reperfusion. The vehicle and GTT were administered to mice 12 h prior to surgery to induce ischemia.

No death occurred in sham-operated mice. Treatment with vehicle or GTT had no effect on survival of control or sham-operated mice. A number representing 79% of vehicle-treated mice survived the first 24 h of reperfusion following bilateral renal ischemia. By day 7 of reperfusion, 70% of mice remained alive (Figure 4). In contrast, 92% of GTT-treated mice subjected to renal ischemia were alive at 24 h post reperfusion and 89% of the animals from this experimental group survived the 7-day period after ischemia (Figure 4). These data show that treatment of mice with GTT prior to renal ischemia/reperfusion-induced injury reduced mortality and enhanced survival during the post reperfusion period.

### 2.4. GTT Administration Promotes Mitochondrial Capacity for Maximum Respiration after Ischemia

**Figure 5:**
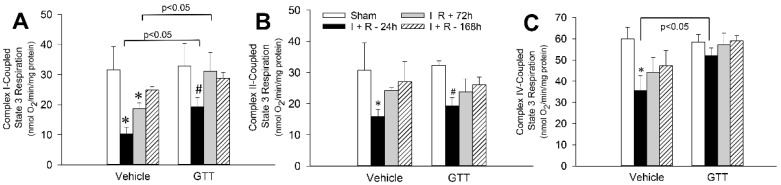
Administration of γ-tocotrienol (GTT) reduces the decreases and promotes the recovery of maximum mitochondrial respiration in renal cortical tissue after ischemia-induced acute kidney injury. State 3 respiration using electron donors to complex I (5 mM glutamate ± 5 mM malate) (**A**), complex II (10 mM succinate ± 0.1 μM rotenone) (**B**), and complex IV (2 mM ascorbic acid ± 1 mM N,N,N′,N′-tetramethyl-p-phenylenediamine) (**C**) in renal cortical mitochondria isolated from mice treated with vehicle (5% Tween 80) or GTT (200 mg/kg b.w.) at 12 h before bilateral renal ischemia (35 min). Mitochondria were isolated from mouse renal cortices at 24, 72, and 168 h after reperfusion. Results are the average ± S.E. of data obtained from six animals in each treatment group. *, # Denote values significantly different (*p* < 0.05) from respective sham kidneys.

State 3 (ADP-stimulated) respiration was measured to assess the maximum oxidative capacity of renal cortical mitochondria. State 3 respiration was not changed by GTT at 12 h after administration (beginning of surgery to induce ischemia) regardless of the substrate used to energize mitochondria (Figure 5). State 3 respiration utilizing glutamate and malate (complex I-coupled respiration) as substrates was decreased to 33% and 60% of sham controls at 24 h and 72 h, respectively, and recovered by day 7 after ischemia in vehicle-treated mice (Figure 5A). GTT administration reduced ischemia-induced decreases in complex I-coupled state 3 respiration to 60% of sham controls at 24 h of reperfusion and promoted full recovery of this function by 72 h after ischemia (Figure 5A). Ischemia also decreased state 3 respiration energized by the oxidation of substrates through complex IV to 59% of vehicle-treated sham controls at 24 h post reperfusion (Figure 5C). GTT prevented this decrease and maintained complex IV-coupled respiration after ischemia at the level present in GTT-treated sham-controls (Figure 5C). In contrast, GTT had no effect on ischemia-induced decreases in complex II-coupled state 3 respiration (oxidation of succinate) during reperfusion (Figure 5B).

These results demonstrate that GTT reduces ischemia-induced decreases in maximum capacity of mitochondria for oxidation of substrates through complexes I and IV of the electron transport chain but plays no role in the decreases of oxidation/reduction reactions that occur at complex II. Furthermore, GTT accelerates recovery of mitochondrial respiration through complex I during reperfusion after ischemia.

### 2.5. GTT Administration Increases Activities of Complexes of the Electron Chain in Cortical Mitochondria of Noninjured Kidneys

Activities of complexes I, III and IV, which translocate H^±^ from the mitochondrial matrix into the intermembrane space and directly contribute to the generation of H^±^ gradient in mitochondria, were followed for 12 h in renal cortical mitochondria after GTT administration to healthy male mice. The goal of these experiments was to determine if GTT administration to mice has an effect on the function or protein levels of these complexes in renal cortical mitochondria in vivo.

#### 2.5.1. NADH:Ubiquinone Oxidoreductase

**Figure 6:**
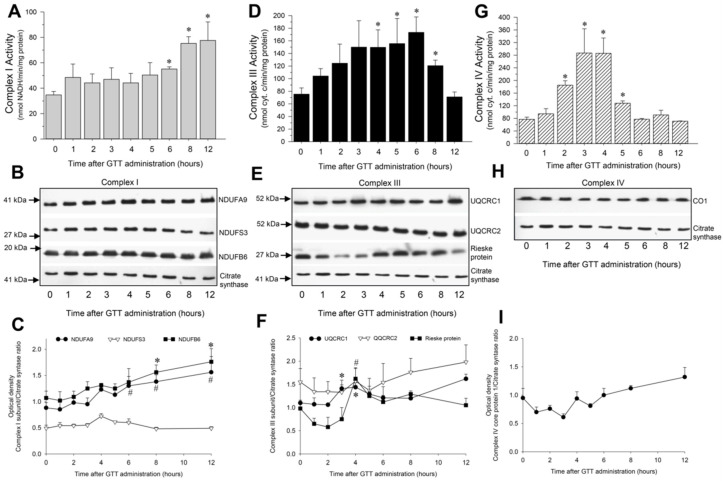
Administration of γ-tocotrienol (GTT, 200 mg/kg b.w.) to mice increases activities of NADH:ubiquinone oxidoreductase (complexes I), ubiquinol-cytochrome c oxidoreductase (complex III), and cytochrome oxidase (complex IV) of the electron transport chain in renal cortical mitochondria. **Upper panel**: Activities of complex I (**A**), complex II (**D**), and complex IV (**G**) in mitochondria isolated from renal cortices of mice at different time points after GTT treatment. Results are the average ± S.E. of data obtained from four animals euthanized per time point after treatment. **Middle panel**: Protein levels of subunits NDUFA9, NDUFS3, and NDUFB6 of complex I (**B**), UQCRC1, UQCRC2, and Rieske protein subunits of complex III (**E**), and CO1 subunit of complex IV (**H**) in mitochondria isolated from renal cortices of mice at different time points after GTT administration. The levels of citrate synthase were used as a loading control in the immunoblots. Immunoblots were carried out as described in Methods. Original blots were stripped off and reprobed using antibodies recognizing other subunits tested in this study. **Lower panel**: Densitometric analysis of immunoblots of subunits of complex I (**C**), complex III (**F**), and complex IV (**I**) in mitochondria isolated from renal cortices of mice at different time points after GTT treatment. The immunoblotting data represent renal cortical mitochondria obtained from three animals euthanized per time point after treatment. * And # represent values significantly different (*p* < 0.05) from respective controls.

In comparison with vehicle-treated mice, the activity of NADH:ubiquinone oxidoreductase (complex I) in renal mitochondria of GTT-treated mice was increased 1.6, 2.2, and 2.6-fold at 6, 8 and 12 h, respectively, after the treatment (Figure 6A). These changes were accompanied by increases in the protein levels of major subunits of complex I, NDUFA9 (39 kDa) and NDUFB6 (17 kDa), in renal cortical mitochondria (Figure 6B,C).

#### 2.5.2. Ubiquinol:Cytochrome c Oxidoreductase

Activity of mitochondrial ubiquinol:cytochrome c oxidoreductase (complex III) was increased 1.6 and 2.5-fold at 3 h and 4 h after GGT administration, respectively, and remained increased until 8 h after the treatment (Figure 6D). The increases in activity of complex III preceded increases in the activity of complex I and returned to control levels by 12 h after the treatment when the activity of complex I was elevated (Figure 6A,D). Increases in the activity of complex III at 3 h and 4 h after GTT administration were associated with elevated mitochondrial levels of the ubiquinol-cytochrome c reductase core protein 1 (UQCRC1) and Rieske subunit of complex III (Figure 6E,F).

#### 2.5.3. Cytochrome Oxidase

Activity of cytochrome oxidase (complex IV) in renal cortical mitochondria increased 2.4-fold at 2 h, reached a maximum at 3 h and 4 h, and returned to control levels by 6 h after GTT administration (Figure 6G). No changes in protein levels of the core protein 1 subunit of cytochrome oxidase occurred during 12 h after GTT administration (Figure 6H,I).

These data show that GTT regulates the activities of complexes I, III, and IV in cortical mitochondria of non-injured kidneys, and that GTT increases protein levels of major subunits forming complexes I and III.

### 2.6. GTT Treatment Preserves Activities of Complexes I and III after Ischemia/Reperfusion Injury

To test whether GTT offers protection against dysfunction of complexes of the electron transport chain in ischemic kidneys, or promotes recovery of these complexes after injury, activities of complexes I, II, III and IV were assessed in renal mitochondria isolated from cortices of noninjured and ischemia-injured kidneys.

#### 2.6.1. NADH:Ubiquinone Oxidoreductase

**Figure 7:**
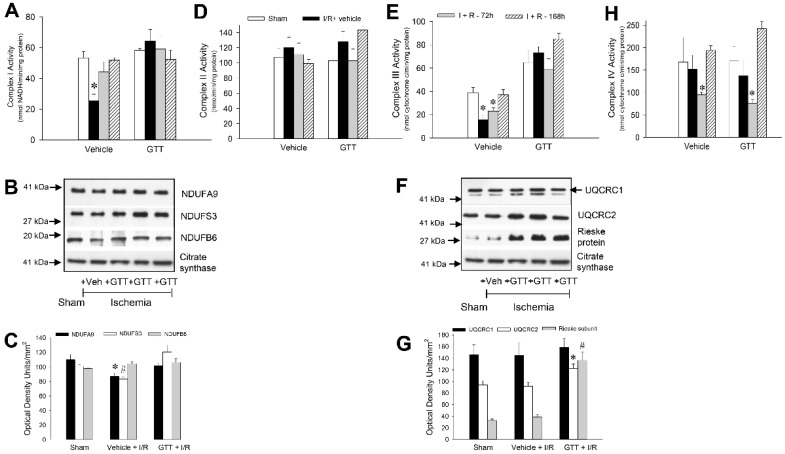
Administration of γ-tocotrienol (GTT) preserves activities of complex I and complex III in renal cortical mitochondria after ischemia-induced acute kidney injury. **Upper panel**: Activities of NADH:Ubiquinone Oxidoreductase (complex I) (**A**), Succinate:Ubiquinone Oxidoreductase (complex II) (**D**), Ubiquinol:Cytochrome c Oxidoreductase (complex III) (**E**), and Cytochrome Oxidase (complex IV) (**H**) in renal cortical mitochondria isolated at 24, 72, and 168 h after ischemia in mice treated with vehicle (5% Tween 80) or GTT (200 mg/kg b.w.) at 12 h before surgery. Results are the average ± S.E. of data obtained from six animals euthanized per time point after treatment. **Middle panel**: Immunoblot analysis of protein levels of subunits NDUFA9, NDUFS3, and NDUFB6 of complex I (**B**), UQCRC1, UQCRC2, and Rieske protein of complex III (**F**) in renal cortical mitochondria isolated at 24 h after ischemia in mice treated with vehicle (5% Tween 80) or GTT (200 mg/kg b.w.) 12 h prior to surgery. The levels of citrate synthase were used as a loading control in the immunoblots. Original blots were stripped off and reprobed using antibodies recognizing other subunits tested in this study. The images shown here are representative of three experiments. **Lower panel**: Densitometric analysis of immunoblots of subunits of complex I (**C**) and complex III (**G**) in renal cortical mitochondria isolated at 24 h after ischemia in mice treated with vehicle (5% Tween 80) or GTT (200 mg/kg b.w.) 12 h prior to surgery. Data represent analysis of immunoblots obtained from three experiments. *, # Represent values significantly different (*p* < 0.05) from respective sham kidneys.

Activity of complex I in renal cortical mitochondria of vehicle-treated mice decreased to 47% and 83% of sham controls at 24 h and 72 h after ischemia, respectively, and fully recovered by day 7 post reperfusion (Figure 7A). In contrast, the activity of complex I was preserved throughout the 7-day recovery period after renal ischemia in mice treated with GTT (Figure 7A). Decreases in the activity of complex I in vehicle-treated mice subjected to ischemia were accompanied by reductions in the protein levels of subunits NDUFA9 (to 78% of sham) and NDUFS3 (to 82% of sham), but not NDUFB6 (Figure 7B,C). The ischemia-induced reductions in protein levels of NDUFA9 and NDUFS3 were prevented by GTT treatment (Figure 7B,C).

#### 2.6.2. Succinate:Ubiquinone Oxidoreductase

The activity of complex II of the respiratory chain in renal mitochondria from vehicle-treated mice was not altered by ischemia at any time point after reperfusion (Figure 7D), which is consistent with our previous reports. Treatment with GTT prior to ischemia had no effect on the activity of complex II in noninjured and ischemia-injured mice kidneys (Figure 7D).

#### 2.6.3. Ubiquinol:Cytochrome c Oxidoreductase

The activity of complex III in renal cortical mitochondria declined to 40% and 60% of sham controls at 24 h and 72 h, respectively, after ischemia in vehicle-treated mice and fully recovered during the 7-day post reperfusion period (Figure 7E). Administration of GTT ameliorated the decreases in complex III activity induced by renal ischemia (Figure 6E). The GTT-offered protection of ischemia-induced decreases in complex III activity was accompanied by elevated mitochondrial levels of core protein 2 (UQCRC2) and the Rieske Fe-S protein subunit of complex III (Figure 7F,G).

#### 2.6.4. Cytochrome Oxidase

In contrast to protective effects of GTT on activities of complexes I and III, GTT had no effect on the decreases and/or recovery of the activity of complex IV after ischemia (Figure 7H).

These results show that complex I and complex III are the major targets of GTT in renal cortical mitochondria and that GTT treatment prevents decreases in activities of these complexes and improves protein levels of major subunits of these complexes after ischemia/reperfusion injury. In contrast, ischemia/reperfusion-induced changes in the activity of complex IV are not alleviated by GTT treatment.

### 2.7. GTT Promotes Activity of ATP Synthase and Ameliorates Decreases in ATP Synthase Activity in Renal Cortical Mitochondria after Ischemia-Induced Injury

**Figure 8:**
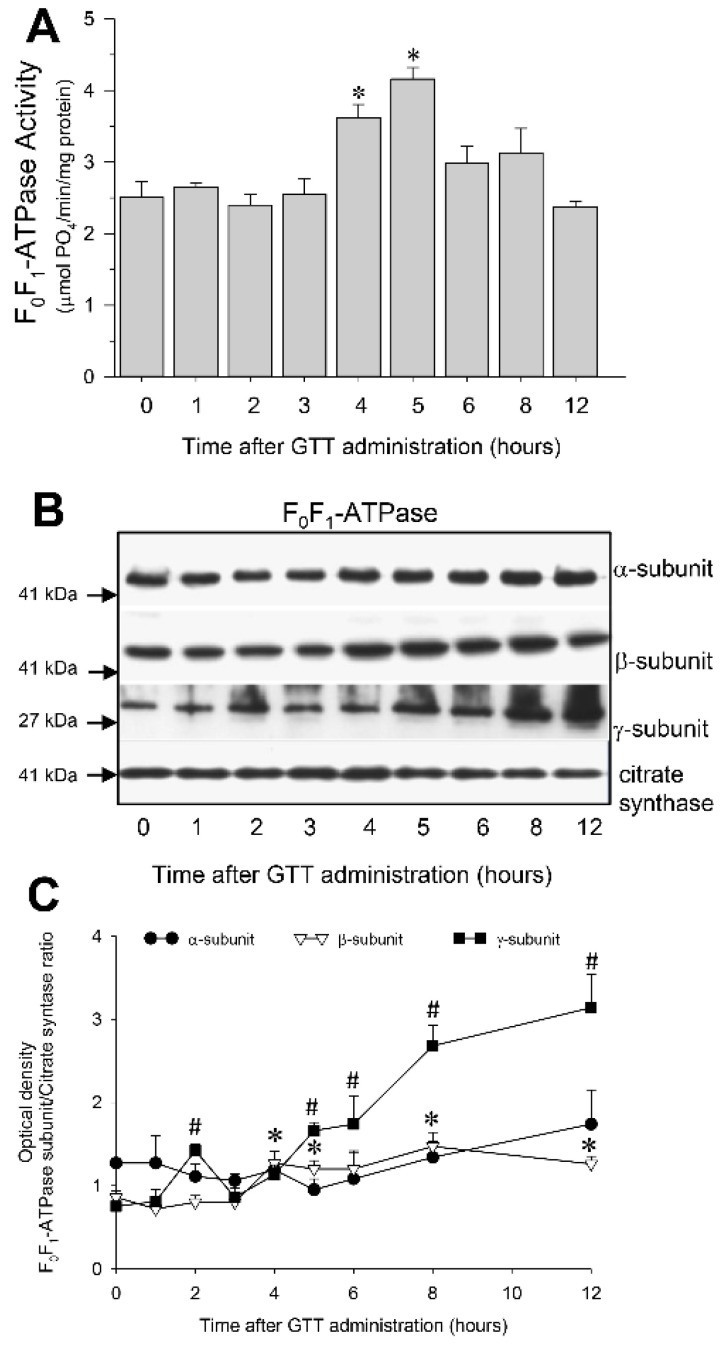
Administration of γ-tocotrienol (GTT, 200 mg/kg b.w.) to mice increases activity of F_0_F_1_-ATPase in renal cortical mitochondria. (**A**) Activity of F_0_F_1_-ATPase in mitochondria isolated from renal cortices of C57BL/6J male mice at different time points after GTT treatment. Results are the average ± S.E. of data obtained from four animals euthanized per time point after treatment. (**B**) Protein levels of subunits α, β catalytic), and γ (central stalk) of F_0_F_1_-ATPase in mitochondria isolated from renal cortices of C57BL/6J male mice at different time points after GTT administration. Immunoblots were carried out as described in Methods. Original blots were stripped off and re-probed using antibodies recognizing other subunits of F_0_F_1_-ATPase. The levels of citrate synthase were used as a loading control. The images are representative of immunoblots of renal cortical mitochondria obtained from three animals euthanized per time point after treatment. (**C**) Densitometric analysis of immunoblots of subunits α, β, and γ of F_0_F_1_-ATPase in mitochondria isolated from renal cortices of C57BL/6J male mice at different time points after GTT treatment. The data represent immunoblots of renal cortical mitochondria obtained from three animals euthanized per time point after treatment. * And # represent values significantly different (*p* < 0.05) from respective controls.

One of the objectives of this study was to test if GTT administration protects against ischemia-induced decreases in F_0_F_1_-ATPase activity or promotes recovery of F_0_F_1_-ATPase after AKI. The activity of F_0_F_1_-ATPase in renal cortical mitochondria of non-injured kidneys was increased 1.5 and 1.6-fold at 4 h and 5 h, respectively, after administration of GTT to mice (Figure 8A). These changes were accompanied by increases in protein levels of the catalytic β subunit and the central γ “stalk” subunit of F_0_F_1_-ATPase complex (Figure 8B,C).

**Figure 9:**
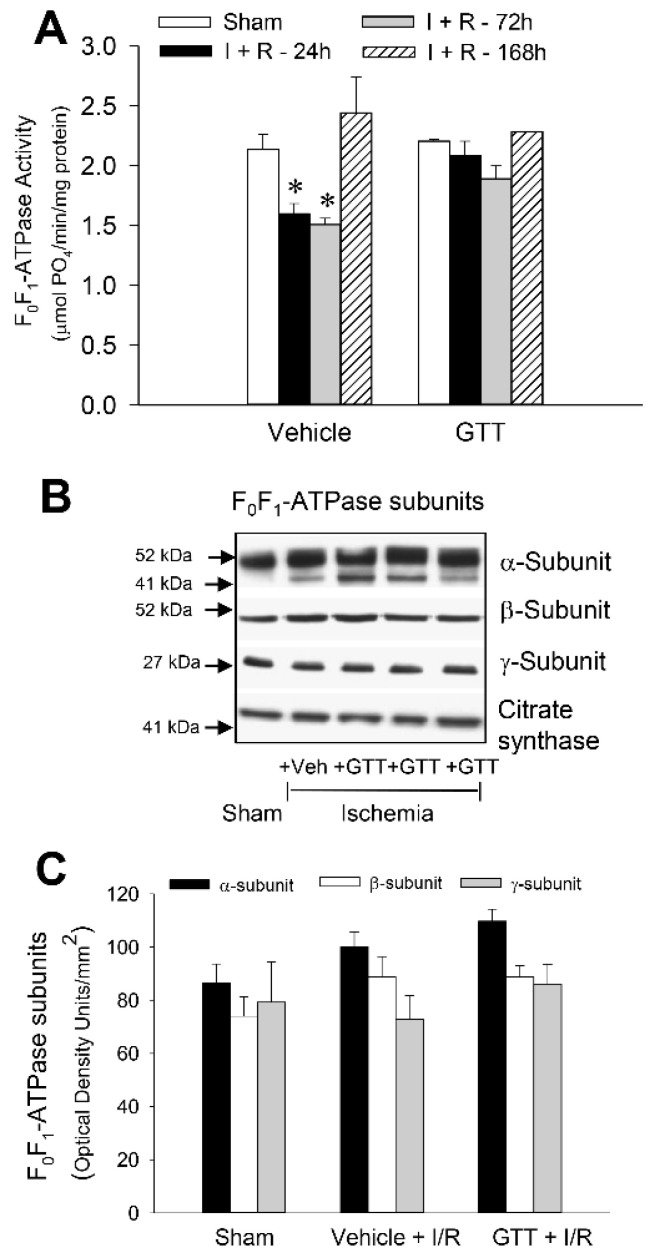
Administration of γ-tocotrienol (GTT) preserves activity of F_0_F_1_-ATPase in renal cortical mitochondria after ischemia-induced acute kidney injury. (**A**) Activity of F_0_F_1_-ATPase in renal cortical mitochondria isolated at 24, 72, and 168 h after ischemia in mice treated with vehicle (5% Tween 80) or GTT (200 mg/kg b.w.) at 12 h before surgery. Results are the average ± S.E. of data obtained from seven animals euthanized per time point after treatment. (**B**) Immunoblot analysis of protein levels of subunits α, β, and γ of F_0_F_1_-ATPase in renal cortical mitochondria isolated at 24 h after ischemia in mice treated with vehicle (5% Tween 80) or GTT (200 mg/kg b.w.) 12 h prior to surgery. The levels of citrate synthase were used as a loading control in the immunoblots, which were carried out as described in Methods. Original blots were stripped off and re-probed using antibodies recognizing other subunits of F_0_F_1_-ATPase. The images shown here are representative of three experiments. (**C**) Densitometric analysis of immunoblots of subunits α, β, and γ of F_0_F_1_-ATPase in renal cortical mitochondria isolated at 24 h after ischemia in mice treated with the vehicle (5% Tween 80) or GTT (200 mg/kg b.w.) 12 h prior to surgery. The data represent analysis of immunoblots obtained from 3 experiments. * Represents values significantly different (*p* < 0.05) from respective sham kidneys.

Ischemia decreased F_0_F_1_-ATPase activity in renal mitochondria of vehicle-treated mice to 50% of sham controls at 72 h and recovered by day 7 post-reperfusion (Figure 9A). This decrease was not accompanied by changes in protein levels of α, β or γ subunits of F_0_F_1_-ATPase in renal cortical mitochondria (Figure 9B,C). In contrast, no decrease in the activity of F_0_F_1_-ATPase occurred in mice treated with GTT before ischemia-induced AKI at any time point studied (Figure 9A). These data show that treatment of mice with GTT prevents decreases in the activity of F_0_F_1_-ATPase after ischemic injury in the kidney and that GTT-offered protection is not through changes in protein levels of F_0_F_1_-ATPase subunits that form the catalytic F_1_ domain of this enzymatic complex.

### 2.8. GTT Administration Maintains Renal ATP Content after Ischemic Injury

ATP content in the renal cortex was assessed to test whether the enhancements in state 3 respiration and the activities of complexes I and III, and F_0_F_1_-ATPase in ischemic kidneys of mice treated with GTT lead to improved levels of renal cortical ATP content. The slices of renal cortex were harvested immediately after animals were sacrificed and the tissue proteins were instantaneously denatured in ice-cold perchloric acid to prevent ATP utilization and hydrolysis.

**Figure 10:**
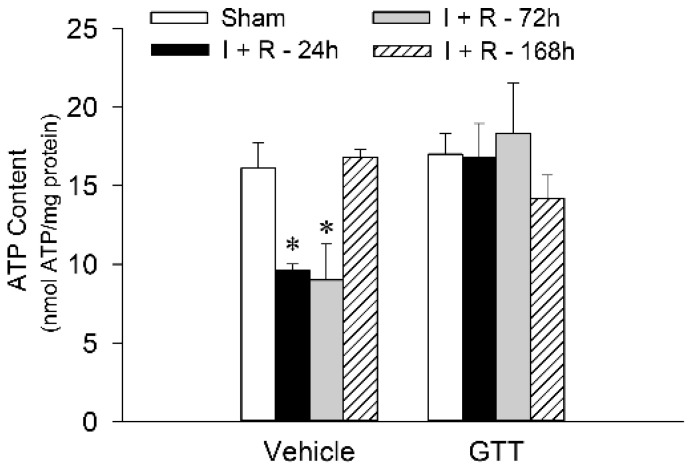
Administration of γ-tocotrienol (GTT) preserves ATP content in renal cortex after ischemia-induced acute kidney injury. The content of ATP was assessed in liquid nitrogen snap-frozen renal cortices dissected immediately after euthanasia from kidneys harvested at 24 h, 72 h, and 168 h after bilateral ischemia (35 min) in mice treated with the vehicle (5% Tween 80) or GTT (200 mg/kg b.w.) 12 h prior to surgery. Results are the average ± S.E. of data obtained from seven animals euthanized per time point after ischemia. * Represents values significantly different (*p* < 0.05) from respective sham-operated controls.

Renal cortical ATP levels in mice treated with the vehicle declined to 60% and 55% of sham controls at 24 h and 72 h, respectively, after ischemia/reperfusion (9.6 ± 0.4 and 9.0 ± 2.2 nmol/mg protein at 24 h and 72 h, respectively, after ischemia vs. 16.1 ± 1.6 nmol/mg protein in renal cortical tissue of sham controls) (Figure 10). ATP content in renal cortex of vehicle-treated mice recovered by day 7 after ischemia (Figure 10). Administration of GTT had no effect on ATP content in cortices of noninjured kidneys (Figure 10). However, GTT prevented decreases in ATP content in renal cortical tissue at all tested time points after ischemia (Figure 10). These data show that GTT prevents energy deficits and preserves ATP content in the renal cortex of the ischemia-injured kidney.

## 3. Discussion

The kidney has a very high mitochondrial content, and mitochondrial content and oxygen consumption at rest is only lower than that in the heart [37]. Oxidative phosphorylation is the primary mechanism of ATP production and meeting energy demands in the renal cortex. Renal proximal tubules do not generate ATP in glycolysis and are completely dependent on mitochondrial production of ATP. Therefore, mitochondrial dysfunction and ATP deficits are major factors in the initiation of injury in the renal cortex and necrosis of renal proximal tubules. Our current study provides a novel observation that GTT administration to mice preserves ATP levels in the renal cortex, reduces renal tubular necrosis and cast formation, and decreases the number of inflammatory cells after renal ischemia. Further, regeneration of renal proximal tubules increases in animals treated with GTT in comparison with those receiving vehicle prior to ischemia. These data suggest that mitochondrial function and ATP production are preserved in renal proximal tubules from animals that are treated with GTT prior to ischemia, and that mitochondrial proteins involved in respiration and/or oxidative phosphorylation are regulated by GTT.

Although in some cancer cell types, for example neoplastic ±SA mammary epithelial cells, tocotrienol-induced apoptosis occurs independently of mitochondrial stress and loss of integrity [38], the majority of cancer cell types respond to GTT treatment by inducing mitochondrial changes that initiate apoptosis [39]. GTT decreases the levels of the NDUFB8 subunit of complex I and SDHB subunit of complex II of the mitochondrial electron transfer chain, inhibits oxidative phosphorylation, increases generation of reactive oxygen species, and induces apoptosis in gastric adenocarcinoma cells [16]. In contrast, GTT has little effect on the COX4I1 subunit of complex IV and the α subunit of ATP synthase. These results from neoplastic cells suggest that mitochondrial respiratory chain is a target for GTT [16].

Interestingly, the effects of GTT on mitochondria in nonmalignant cells are opposite to those in cancer cells. GTT administration leads to protection of cells and organs from different types of injury including irradiation-induced and toxicant-induced injury. For example, mitochondrial mass, respiration and coupling, citrate synthase activity, mitochondrial membrane potential, and the levels of ATP, cardiolipin and respiratory chain complexes I and II, are enhanced in the brains of animals fed with stabilized rice bran extract containing tocotrienols, or the supplementation of their diets with GTT and δ-tocotrienol [40,41]. GTT demonstrates antioxidative efficacy, attenuates emphysematous lesions, improves lung function, and protects against emphysema in chronic obstructive pulmonary disease [25]. Our previous report demonstrated that GTT is taken up by RPT cells in primary culture and acts as a very potent protectant of mitochondrial function, improves mitochondrial respiration, reduces cellular energy deficits, and prevents oxidant-induced death of these cells in vitro [34]. The protective concentrations of GTT in renal cells in vitro are much lower (5–10 μM) than the apoptosis-induced concentrations of GTT used in cultured cancer cells (30–80 μM) [16,34]. GTT concentrations higher than 20 μM have a detrimental effect on mitochondrial functions, and 50 μM GTT is cytotoxic in RPT cells in vitro ([34] and unpublished data). Therefore, a different range of GTT concentrations used in different in vitro studies can explain the protective or cytotoxic actions of GTT reported in different publications.

Our previous in vitro study provided enough data to test two hypotheses: (1) GTT protects the kidney from AKI or improves kidney recovery from AKI in vivo, and (2) GTT targets mitochondrial electron transport chain and oxidative phosphorylation to decrease energy deficits in the renal cortex in vivo. Previous observations in mice have shown that the kidney takes up and accumulates GTT provided in the diet and that the content of GTT in the kidney is similar to that in the heart and liver [4]. Our present in vivo study in mice provides a novel observation that GTT reduces dysfunction of the electron transport chain and accelerates its recovery in renal cortical mitochondria after ischemia. Respiration coupled to oxidation of substrates by NADH-ubiquinone oxidoreductase (complex I) is the subject of the protective actions of GTT in vivo, whereas respiration coupled to succinate-ubiquinone oxidoreductase (complex II) is not improved by GTT. Thus, our study focused on the major protein complexes that mediate electron transport chain reactions and respiration in mitochondria. Our results show that complexes I and III of the electron transport chain are major targets of GTT in renal cortical mitochondria, and that GTT administration to mice enhances the activities of complexes I and III in isolated renal cortical mitochondria. Our data contrast with the downregulation of protein levels and activities of complexes I and II by GTT reported in neoplastic cells in vitro [16]. It is likely that the higher concentrations of GTT used by Wang and colleagues (30–80 μM) were detrimental to mitochondrial proteins and their functions.

Interestingly, the maximum activity of each complex occurred at different time points after GTT exposure. Activity of complex IV reached a maximum within 3–4 h and subsided within 6 h after GTT administration. These increases were not accompanied by changes in protein levels of complex IV. Complex III reached maximum activity later than complex IV i.e., between 4 and 6 h. The increases in complex III activity were accompanied by elevation in protein levels of the Core Protein 1 (UQCRC1) and Rieske Fe-S protein of the complex. However, increases in the activity of complex III in renal cortical mitochondria were transient and returned to control levels before ischemic injury was induced. In contrast, the activity of complex I did not reach a maximum until 8 h after GTT treatment and remained elevated at 12 h post injection when the ischemia surgery began. The increases in complex I activity were preceded by a gradual increase in protein levels of NDUFA9 and NDUFB6 subunits of complex I. Protein levels of NDUFA9 and NDUFB6 remained at an elevated level at 12 h after GTT administration. This increase coincided with the maximum uptake of GTT in RPT cells at 12 h after treatment, as we reported previously [34]. NDUFA9 is a core subunit of complex I and contains an NADP(H)-binding site. NDUFA9 is critical for the stability of complex I because of its association with the essential core ND3 subunit of the orthogonal arm of complex I buried in the inner membrane [42]. Stable association of NDUFA9 and ND3 subunits is critical for anchoring the peripheral (NADH oxidizing) arm of complex I to the inner mitochondrial membrane and for the normal function of complex I. Decreased levels or defects in NDUFA9 associations with other subunits are thought to cause complex I instability, which results in different developmental abnormalities and neonatal mortality [43,44].

The accessory NDUFB6 subunit of complex I is not directly involved in catalysis, but is required for the normal function of the electron transfer chain [43]. Deficiency or dysfunction of this subunit is associated with mitochondrial myopathy, encephalopathy, lactic acidosis, stroke-like episodes, and mitochondrial complex I deficiency [45]. NDUFB6 has a highly conserved two-domain structure with a sequence of positively charged residues in the N-terminal hydrophobic domain that are important for protein–protein interactions. It has been proposed that the hydrophobic domain of NDUFB6 anchors the peripheral NADH dehydrogenase arm to the inner mitochondrial membrane and stabilizes the functional complex in the inner mitochondrial membrane [46]. Therefore, increased protein levels of NDUFA9 and NDUFB6 in response to GTT treatment may improve complex I activity by stabilizing associations of the NADH oxidizing arm domain with the H^±^-pumping domain of complex I embedded in the inner mitochondrial membrane. A previous report showed that GTT changes the expression of mRNA coding several subunits of complexes I and II, but not complexes III and IV in cancer cells in vitro [16]. Still, it is unknown if GTT increases the transcription and translation of these subunits in our in vivo model in the kidney or decreases their degradation by stabilizing them within the inner mitochondrial membrane. Both actions could lead to increased levels of NDUFA9 and NDUFB6. Nevertheless, our findings indicate that GTT regulates protein levels of important subunits of complex I that play a role in the assembly and/or stability of this complex, and ultimately, improved activity. Stabilization of subunits forming complex I by GTT may explain not only the increase in the activity of this complex in kidneys of nonischemic mice, but also the protective actions of this compound against decreases in the activity of complex I after ischemia. Renal cortical mitochondria isolated from mice treated with GTT before ischemia had unchanged activity and protein levels of all tested subunits of complex I after ischemia. In contrast, mitochondria isolated from mice treated with the vehicle had decreased activity of complex I and lower protein levels of NDUFA9 and NDUFS3 subunits at 24 h after ischemia.

Likewise, ischemia decreased the activity of complex III in mitochondria isolated from renal cortices at 24 h and 72 h post reperfusion in mice treated with the vehicle. In contrast, protein levels of the three major subunits of complex III were increased in renal cortical mitochondria of sham mice and unchanged by ischemia. We assessed protein levels of core protein 1 (UQCRC1), core protein 2 (UQCRC2), and Rieske Fe-S protein of complex III because these subunits have been implicated in maintaining the assembly and activity of complex III, and in the response of some tissues to hypoxia and ischemia. Our data demonstrate that increases in protein levels of UQCRC1 and Rieske protein coincide with increases in complex III activity in renal cortex after GTT administration in nonischemic mice. Although the exact functions of the UQCRC1 subunit are not clear, it was shown that decreased levels or dysregulated UQCRC1 resulted in deficient formation and activity of complex III, impaired mitochondrial electron transport chain function, and decreased ATP content in the mouse brain [47]. UQCRC1 was implicated in ischemic tolerance, learning, and memory in the brain. Deficiency in UQCRC1 worsens neurological outcomes after brain ischemia or hypoxia [47]. Interestingly, dysregulation of UQCRC1 has been implicated in kidney transplant rejection [48]. In contrast, upregulation of UQCRC1 levels improves mitochondrial function and prevents endothelial cell death [49]. UQCRC1 overexpression plays a role in cardioprotection, increases the levels of Bcl-2, decreases the levels of Bax and active caspase 3, and reduces apoptosis in H9c2 cardiac cells [50]. UQCRC2 is necessary for the formation of functional complex III and has been implicated in processing of the UQCRFS1 subunit into the mature Rieske Fe-S protein. Disruptions in UQCRC2 have been associated with mitochondrial complex III deficiency nuclear type 5, which results in a variety of disorders including mitochondrial encephalopathy, liver dysfunction, renal tubulopathy, hypoglycemia, and exercise intolerance [51]. The role of UQCRC2 in renal injury is unknown. Our results show that protein levels of UQCRC2 and Rieske Fe-S subunits are increased, and UQCRC1 levels are preserved, in the renal cortex of ischemic kidneys of mice treated with GTT before ischemia. Rieske Fe-S protein is the last incorporated subunit during the assembly of complex III of the electron transport chain, and the presence of this protein is critical for the enzymatic activity of complex III. During the assembly of complex III, the N-terminal domain of Rieske protein remains bound between the two core subunits (UQCRC1 and UQCRC2) of complex III [52]. The improvements in protein levels of UQCRC2 and Rieske Fe-S subunit in kidneys of mice that received GTT were accompanied by the preserved activity of complex III and state 3 respiration in mitochondria after ischemia, whereas these improvements were absent in renal mitochondria isolated from mice that received the vehicle.

GTT administration ameliorated ischemia-induced decreases in respiration coupled to cytochrome oxidase (complex IV) at 24 h after reperfusion. However, the mechanism of this protection was not through direct improvement of the function of complex IV. The decreases in the activity of complex IV in mitochondria isolated from renal cortices were equivalent in vehicle- and GTT-treated mice at 24 h post reperfusion. Although GTT transiently increased the activity of complex IV after GTT administration in nonischemic mice, GTT was ineffective in preserving the activity of complex IV after ischemic injury. These data suggest that either complex IV is not a target of GTT in the kidney, or that the effects of GTT on complex IV are short lived in comparison with those observed for complexes I and III. Our results are consistent with previous report that GTT has no effect on complex IV in cancer cells [16].

GTT administration in nonischemic mice resulted in a gradual increase in protein levels of the catalytic (β) subunit and the central stalk (γ) subunit that links the α and β subunits of the F1 domain of F_0_F_1_-ATPase. However, GTT had no effect on the levels of the α-subunit of F_0_F_1_-ATPase. Concurrently, the activity of this enzyme increased between 4 and 5 h after GTT administration, but the increase was transient and F_0_F_1_-ATPase activity subsided within 6 h after GTT treatment in nonischemic mice despite increased levels of the β and γ subunits. This could be explained by a feedback response of the regulatory enzymes of the pathways of oxidative metabolism to increased production of ATP and subsequent downregulation of F_0_F_1_-ATPase activity. However, in mice that were treated with GTT, increased protein levels of subunits making up the F1 (catalytic) domain of ATP synthase were accompanied by preserved activity of F_0_F_1_-ATPase and ATP levels at 24 h after ischemia. In contrast, F_0_F_1_-ATPase activity and ATP content were significantly decreased in vehicle-treated mice at the same time after ischemia. Our results are consistent with the published observations in cancer cells that GTT had very little effect on the levels of the α (ATP5F1A) subunit of F_0_F_1_-ATPase [16].

The results of our current study in vivo are consistent with the findings reported in our previous study in primary cultures of renal proximal tubular cells (RPTC) in vitro [34]. In that study, we demonstrated that GTT protects RPTC against mitochondrial dysfunction and RPTC injury and death caused by oxidants [34]. Specifically, GTT blocked production of the reactive oxygen species, ameliorated decreases in state 3 and oligomycin-sensitive respirations, and maintained the respiratory control ratio and mitochondrial membrane potential in oxidant-injured RPTC [34]. Further, GTT maintained F_0_F_1_-ATPase activity and intracellular ATP content and blocked decreased RPTC lysis and death after injury [34]. The results of the current in vivo study are consistent with the previous findings and provide more insight into GTT-initiated mechanisms of the improved mitochondrial function in injured kidney.

## 4. Materials and Methods

### 4.1. Animals

All animal procedures were carried out in accordance with the Public Health Service and NIH Policy on Humane Care and Use of Laboratory Animals. These procedures were ethically reviewed and approved by the Institutional Animal Care and Use Committee at the University of Arkansas for Medical Sciences. C57BL/6J male mice were supplied by The Jackson Laboratory (Bar Harbor, ME, USA). The mice were housed at constant room temperature of 20 °C and 12 h light/dark cycle with free access to standard diet and water.

### 4.2. γ-Tocotrienol Administration

γ-Tocotrienol (GTT) was purchased (Cayman Chemical, Ann Arbor, MI, USA) as a solution (1 mg/mL) in 100% ethanol and stored at −20 °C until used. An aliquot of GTT was transferred to a light blocking amber tube and ethanol was quickly evaporated under a stream of 100% nitrogen. GTT remaining after solvent evaporation was resuspended in a sterile solution of Tween-80 (5%) in double distilled water, vigorously vortexed, and injected subcutaneously into mice. In preliminary experiments, we tested two different doses of GTT, 100 mg/kg and 200 mg/kg body weight (b.w.). Although the dose of 100 mg/kg b.w. had a partial protective effect on kidney morphology after ischemia, it was not as effective as the higher dose of 200 mg/kg b.w. This is consistent with the GTT dose that offers protection against intestinal injury after total body irradiation in mice [23,24].

Initial experiments tested whether GTT administration had an effect on the activities and protein levels of major complexes of the oxidative phosphorylation in the kidneys of control mice. Thirty two C57BL/6J male mice were injected with the sterile solution of GTT (200 mg/kg b.w. in 5% Tween-80) and kidneys were harvested at 1, 2, 3, 4, 5, 6, 8 and 12 h after the injection to determine if GTT had an effect on the activities and protein levels of complexes of the respiratory chain and F_0_F_1_-ATPase in isolated renal cortical mitochondria from uninjured kidneys. Control mice received an equivalent volume of sterile vehicle (5% Tween-80 in sterile water).

In the subsequent set of experiments, mice were injected with the sterile GTT suspension (200 mg/kg b.w.) or vehicle (5% Tween-80) at 12 h prior to bilateral renal ischemia, and blood and kidneys were harvested on days 1, 3 and 7 after reperfusion to determine kidney functions and morphology, mitochondrial functions, activities and protein levels of complexes of the respiratory chain and ATP synthase, and renal cortical ATP content. Four experimental conditions were tested in each experiment: (1) male mice treated with the vehicle (5% Tween 80) and subjected to sham surgery; (2) male mice treated with the vehicle and subjected to surgery to induce bilateral renal ischemia; (3) male mice treated with GTT (200 mg/kg b.w. suspended in the vehicle) and subjected to sham surgery, and (4) male mice treated with GTT (200 mg/kg b.w. suspended in the vehicle) and subjected to the surgery to induce bilateral renal ischemia. The total number of animals used was: (1) 16 male mice treated with the vehicle (5% Tween 80) and subjected to sham surgery; (2) 42 male mice treated with the vehicle and subjected to the surgery to induce bilateral renal ischemia; (3) 16 male mice treated with GTT (200 mg/kg b.w. suspended in the vehicle) and subjected to sham surgery; and (4) 42 male mice treated with GTT (200 mg/kg b.w. suspended in the vehicle) and subjected to the surgery to induce bilateral renal ischemia. The survival of mice in each experimental group was monitored and is reported in the results.

### 4.3. Ischemia/Reperfusion Injury

It has been shown that male mice are more susceptible to ischemic kidney injury when compared with females and that these differences are due to the actions of testosterone [53,54]. Thus, adult (3–4 months old) male mice were used to determine if GTT has protective effects against ischemic injury in the kidney. Mice were anesthetized with sodium pentobarbital (50 mg/kg b.w.; Ovation Pharmaceuticals, Deerfield, IL, USA) and all surgical procedures were performed under sterile conditions. Following a midline incision of the abdomen, kidneys were exposed and decapsulated, and both renal hila were clamped for 35 min with small vascular clamps to induce bilateral renal ischemia as described previously [55,56,57,58]. While renal arteries of both kidneys were clamped, mice were placed on a heating pad that maintained a temperature of 37 °C. During that time, approximately 0.5 mL sterile saline was applied intraperitoneally to prevent fluid loss and dehydration. After 35 min of ischemia, clamps were released, both kidneys were reperfused, checked for any perfusion problems, and the animal abdomen was closed using 4-0 silk suture. Sham operations involved all surgical procedures and kidney manipulations without inducing ischemia. The mice were returned to their cages, allowed free access to food and water, and given buprenorphine subcutaneously as needed for the first 72 h post reperfusion to control pain. Animals were closely monitored for 168 h (7 days) after surgery. On days 1, 3, and 7 after ischemia, animals were euthanized, and blood and kidneys were harvested to assess the endpoints, which included renal functions, tissue morphology, mitochondrial functions and enzyme activities, and ATP content in the renal cortex.

### 4.4. Assessment of Renal Morphology

Immediately after harvesting, kidneys were fixed in 4% neutral-buffered formaldehyde for at least 24 h and embedded in paraffin. Thin (4–8 μm) tissue sections were cut and stained with hematoxylin-eosin and periodic acid Schiff (PAS) as described previously [55,56]. Images were acquired using Nikon Eclipse E800 light microscope and Nikon Plan Apo objective. The following histologic criteria were used to assess the kidney morphology and determine tissue damage: tubular necrosis, brush border loss, red blood cell extravasation, tubular dilatation, tubular cast formation, degeneration and inflammation. Degeneration was scored by assessing cell swelling, formation of vacuoles, the presence of PAS-positive granules in cytoplasm due to injury to the membranes, disruption of ion transport, and accumulation of intracellular water, lipids, and/or proteins/lipoproteins. Regeneration was scored in the tubules that underwent necrosis and were relined by new undifferentiated epithelial cells. Inflammation was determined based on the density and numbers of inflammatory cells throughout the kidney. A score in each criterion was given depending on the extent of the changes. These above-mentioned markers were evaluated on a scale of 0 to 4: absent (0), mild (1), moderate (2), severe (3), and very severe (4). Kidneys obtained from 3–4 different mice from each experimental group and time point (days 1, 3, and 7 post reperfusion) were used to assess renal morphology.

### 4.5. Plasma Levels of Creatinine

Plasma levels of creatinine were used as a marker of renal function. Blood was drawn while mice were euthanized, spun down and plasma was used to determine creatinine concentration using the Creatinine Reagent Set from Biotron Diagnostics (Hemet, CA, USA) and the manufacturer’s protocol, as reported previously [56,57,58]. Plasma creatinine levels were determined in all mice used in the four experimental groups described on page 3.

### 4.6. Isolation of Mitochondria

Immediately after mice were euthanized, both kidneys were harvested, submerged in an ice-cold isolation buffer (10 mM Hepes, 225 mM mannitol, 75 mM sucrose, 2 mM EGTA, and 0.1% BSA, fatty acid free, pH 7.4) and dissected on ice as described previously [56,57,58]. Cortical tissue separated from the kidneys was homogenized in ice-cold isolation buffer and the homogenate was spun down at 1000× *g* 5 min at 4 °C. The supernatant resulting from this centrifugation was spun down at 15,000× *g* 15 min at 4 °C to obtain a mitochondrial pellet. The mitochondria were washed twice using the isolation buffer and centrifuged at 15,000× *g* 10 min (4 °C) after each washing. The final mitochondrial pellet was resuspended in the isolation buffer and used to assess respiration, the activities of complexes of the electron transport chain and F_0_F_1_-ATPase, and immunoblotting.

### 4.7. State 3 Respiration

State 3 respiration was assessed in an assay buffer (20 mM Hepes, 137 mM KCl, 2 mM KH_2_PO_4_, 0.5 mM EGTA, 5 mM MgCl_2_, pH 7.4) by following mitochondrial consumption of O_2_ as described previously [56]. Glutamate (5 mM) and malate (5 mM) were used to measure respiration coupled to complex I of the electron transport chain. Succinate (10 mM) in the presence of 0.1 μM rotenone (inhibitor of complex I) served as a substrate to determine respiration coupled to complex II of the electron transport chain. State 3 respiration coupled to complex IV (cytochrome oxidase) was assessed by using 2 mM ascorbic acid ± 1 mM N,N,N′,N′-tetramethyl-p-phenylenediamine as electron donors. State 3 respiration was initiated by adding ADP (0.4 mM). State 4 respiration was determined after adding oligomycin (2.5 μg/mL; Calbiochem, San Diego, CA, USA) to mitochondria respiring at state 3. The reagents used to determine state 3 respiration and activities of complexes of the electron transport chain were purchased from Sigma-Aldrich (St. Louis, MO, USA).

### 4.8. Activities of Respiratory Complexes

Activities of complexes of the electron transport chain were measured in isolated mitochondria in vitro, as described previously [56,59]. Complex I (NADH:Ubiquinone Oxidoreductase) activity was determined in mitochondria suspended in the assay buffer consisting of 10 mM KH_2_PO_4_, 5 mM MgCl_2_, and 0.25% BSA; at pH 7.2. In addition to mitochondria, the assay mixture contained 62.5 μM ubiquinone (as the electron carrier), antimycin A (2 μg/mL) to inhibit complex III, and NADH (0.25 mM) as a substrate. Oxidation of NADH by complex I was measured spectrophotometrically for 3 min at 340 nm and 30 °C, was followed by addition of rotenone (10 μg/mL), and the measurement continued for another 2 min. Complex I activity was expressed as the rotenone-sensitive oxidation of NADH.

Complex II (Succinate:Ubiquinone Oxidoreductase) activity was measured in freshly isolated mitochondria suspended in the assay buffer containing 20 mM succinate as the oxidative substrate, 0.25% BSA, antimycin A (2 μg/mL), rotenone (10 μg/mL), ubiquinone (62.5 μM), and dichlorophenolindophenol (0.25 mM) as the electron acceptor. The reduction of dichlorophenolindophenol was followed spectrophotometrically for 3 min at 590 nm and 30 °C.

Complex III (Ubiquinol:Cytochrome c Oxidoreductase) activity was determined in freshly isolated mitochondria suspended in the assay buffer containing 0.25% BSA, rotenone (10 μg/mL), KCN (0.24 mM) to inhibit complex IV, decylubiquinol (50 μM) as a substrate, and cytochrome c (60 μM) as the electron acceptor. The reduction of cytochrome c was followed spectrophotometrically for 3 min at 550 nm and 30 °C. Antimycin A (2 μg/mL) was added at the end of the measurement and the absorbance was followed for another 2 min. The activity of complex III was expressed as the antimycin A-sensitive reduction of cytochrome c.

Complex IV (Cytochrome Oxidase) activity was determined in freshly isolated mitochondria suspended in the assay buffer containing 10% BSA, reduced cytochrome c (90 μM), and antimycin A (2 μg/mL). The activity of complex IV was assessed by following the oxidation of reduced cytochrome c at 550 nm (30 °C) in the absence and presence of KCN (0.24 mM). Complex IV activity was calculated as KCN-sensitive oxidation of cytochrome c.

### 4.9. Activity of F_0_F_1_-ATPase

ATPase activity of ATP synthase was determined in freshly isolated mitochondria by measuring hydrolysis of ATP as previously described [59,60,61]. After isolation, the mitochondrial pellet was suspended in an ice-cold buffer (10 mM KH_2_PO_4_, 5 mM MgCl_2_, pH 7.2). Hydrolysis of ATP was measured at 31 °C in a Tris-HCl buffer (pH 8.2) containing 200 mM KCl, 2 mM MgCl_2_, and 5 mM ATP in the absence and presence of oligomycin (10 μg/mL). The oligomycin-sensitive ATPase activity was calculated and reported. All reagents used to determine activity F_0_F_1_-ATPase were purchased from Sigma-Aldrich (St. Louis, MO, USA).

### 4.10. Tissue ATP Content

Renal cortical tissue slices were dissected immediately in ice-cold isolation buffer after animal euthanasia, placed into an ice cold 3% perchloric acid, snap-frozen in liquid nitrogen, and stored at −80 °C until analyzed. Frozen samples were thawed, sonicated in 3% perchloric acid on ice, and spun down at 10,000× *g* for 1 min at 4 °C. The supernatant was collected, placed on ice, neutralized to pH of 7.4 with KOH, and used to determine the content of ATP. ATP concentration in neutralized supernatant was measured by the luciferase luminescence method using Bioluminescence Assay Kit HS II (Roche, Mannheim, Germany) and the manufacturer’s protocol as described previously [61,62]. The pellets containing denatured tissue proteins were solubilized in 100 mM Tris-HCl buffer, pH 7.4, containing 150 mM NaCl buffer and 0.05% Triton X-100, sonicated on ice, and used to determine protein concentration in samples. ATP content was expressed as nmoles of ATP per mg of tissue protein.

### 4.11. Immunoblotting

Immunoblot analysis was used to assess protein levels of subunits of complexes I, III and IV, and subunits of F_0_F_1_-ATPase in renal cortical mitochondria using 20 μg of mitochondrial protein, as described previously [57,61]. Antibodies against NDUFA9, NDUFS3, and NDUFB7 subunits of complex I (Cat #A21344, A21343, and 21359) were purchased from Molecular Probes/ThermoFisher Scientific (Waltham, MA, USA). Antibodies against α- and γ-subunits of F_0_F_1_-ATPase (Cat #ab14748 and #ab119686, respectively) and the core protein 1 (UQCRC1), core protein 2 (UQCRC2), and Rieske protein subunits of complex III (Cat #ab110252, #ab14745, and #ab14746, respectively) were supplied by Abcam (Cambridge, MA, USA). Antibodies against β-subunit of F_0_F_1_-ATPase (Cat #A21351) were supplied by Life Technologies Corporation (Carlsbad, CA, USA). Cytochrome oxidase subunit I antibody was supplied by Santa Cruz Biotechnology (Santa Cruz, CA, USA, Cat #sc-58347). Citrate synthase levels served as the loading control using the citrate synthase antibody (Cat #D7V8B) supplied by Cell Signaling Technology (Danvers, MA, USA).

### 4.12. Protein Concentration

Protein concentration was determined using bicinchoninic acid assay with bovine serum albumin as the standard, as described previously.

### 4.13. Statistical Analysis

Data are shown as means ± S.E. Experimental groups were analyzed for significant changes by the two-tailed Student t test for independent samples or by ANOVA. Multiple means were compared using Fisher’s protected least significance difference (LSD) test with a level of significance of *p* < 0.05.

## 5. Conclusions

The better known, described, and accepted functions of tocotrienols are related to their antioxidant (inhibition of oxidative and nitrosative stress) and anti-inflammatory properties. However, antioxidants alone have been ineffective in reducing or preventing acute kidney injury, its complications, and consequences. Thus, the protective actions of γ-tocotrienol treatment to maintain the function of mitochondria and tissue ATP levels in ischemia-injured kidneys are not due to antioxidant and/or anti-inflammatory effects, only. The results of this study show that GTT may prove therapeutically valuable in preventing renal injury in vivo. The current study supports preventive application of GTT in clinical settings. Acute kidney injury often occurs in clinical/hospital settings as a consequence of major surgeries, trauma, or diagnostic procedures that are likely to produce kidney injury. Administration(s) of GTT prior to these events and procedures could be an effective preventive approach to protect kidneys in clinical situations that have high probability to lead to AKI, particularly in the older population that is more prone to developing AKI. It is noteworthy to mention that GTT administration has not been found toxic to other organs or nonmalignant tissues. Furthermore, our preliminary data (not included in this report) suggest that administration of GTT also offers significant degrees of mitochondrial and renal protection if administered at the onset of reperfusion. This suggests that GTT may be effective not only as a preventive therapy, but also as a therapy supporting kidney recovery after kidney injury has occurred, possibly in community settings. Our study suggests that physiological role of GTT in human health deserves further investigation and that the mechanisms of protective properties of this lesser-known form of vitamin E represent a research direction that is clinically relevant.

Our novel observations allow us to conclude that administration of γ-tocotrienol provides significant protection of morphology and functions in kidneys subjected to ischemic injury and diminishes AKI. Further, γ-tocotrienol administration markedly reduces mortality associated with AKI. Our data show that γ-tocotrienol preserves mitochondrial functions involved in ATP production in the kidneys subjected to ischemia and that mitochondrial complexes I and III of the electron transport chain are the major targets of this compound. γ-Tocotrienol prevents ischemia-induced decreases in protein levels of important subunits making up complexes I and III, preserves their activities, and promotes mitochondrial respiration coupled to oxidation of substrates through these two complexes after ischemia. Finally, administration of γ-tocotrienol preserves ATP content and prevents energy deficits in renal cortical tissue after ischemia. Our study also suggests that γ-tocotrienol could be used as a therapeutic approach to minimize AKI in hospitalized patients and accelerate kidney recovery if AKI has occurred.

## Figures and Tables

**Table 1:** Changes in kidney morphology in mice treated with vehicle (5% Tween-80) and γ-tocotrienol (200 mg/kg b.w.) during recovery after bilateral renal ischemia. The following histologic criteria were used to assess morphological damage to the kidney: tubular necrosis, brush border loss, tubular cast formation, degeneration, inflammation, and regeneration. These parameters were evaluated on a scale of 0–4: not present (0), mild (1), moderate (2), severe (3), and very severe (4). Results are presented as average ± S.E. of data obtained from kidney sections from vehicle- and GTT-treated mice. Each time point represents data obtained from kidneys harvested from 3 male mice. * Values significantly different (*p* < 0.05) from vehicle-treated mice.

	Tubular Necrosis	Brush Border Loss	Cast Formation	Tubular Dilation	Inflammatory Cells	Interstitial Edema	Degeneration	Regeneration	RBC Extravasation
Sham	0.0 ± 0.0	0.0 ± 0.0	0.5 ± 0.3	0.0 ± 0.0	0.0 ± 0.0	0.0 ± 0.0	0.0 ± 0.0	0.0 ± 0.0	1.0 ± 0.0
**Vehicle + Ischemia/Reperfusion**									
Day 1	3.7 ± 0.2	2.9 ± 0.5	3.2 ± 0.5	2.5 ± 0.4	2.5 ± 0.3	0.0 ± 0.0	1.7 ± 0.3	0.0 ± 0.0	2.3 ± 0.7
Day 3	2.9 ± 0.7	2.3 ± 0.7	3.3 ± 0.8	2.4 ± 0.9	0.9 ± 0.7	1.0 ± 0.4	1.0 ± 0.6	1.8 ± 0.8	3.3 ± 0.5
Day 7	3.3 ± 0.2	2.0 ± 0.6	3.3 ± 0.3	2.4 ± 0.9	0.9 ± 0.7	1.7 ± 0.7	1.0 ± 0.6	2.3 ± 0.2	2.3 ± 0.9
**γ-Tocotrienol + Ischemia/Reperfusion**									
Day 1	2.6 ± 0.3 *	2.2 ± 0.4	3.0 ± 0.4	2.2 ± 0.2	1.0 ± 0.0 *	0.4 ± 0.2 *	0.8 ± 0.6	2.2 ± 0.6 *	1.9 ± 0.6
Day 3	1.9 ± 0.2	1.1 ± 0.1	2.6 ± 0.2	2.1 ± 0.1	1.0 ± 0.0	0.5 ± 0.3	0.3 ± 0.1	3.1 ± 0.5	1.5 ± 0.2 *
Day 7	1.2 ± 0.2 *	1.3 ± 0.5	1.6 ± 0.5 *	1.0 ± 0.4 *	1.4 ± 0.5	0.8 ± 0.3	0.4 ± 0.3	3.8 ± 0.3 *	0.6 ± 0.5 *

## Data Availability

Data available from the authors upon request.
